# Supplementary material for: Neonatal and Long-Term Prognosis of Monochorionic Diamniotic Pregnancies Complicated by Selective Growth Restriction
Source: Children (Basel). 2022 May 11;9(5):708. doi: 10.3390/children9050708 (PMC9139785; doi:10.3390/children9050708)
Supplement: Supplementary file 1 [file children-09-00708-s001.zip › children-1635628-supplementary.pdf]

Table S1. Comparison of neonatal status between the "diamniotic monochorionic twin pregnancy with selective intrauterine growth restriction" and "single pregnancy with intrauterine growth restriction".

	N	sIUGR	IUGR	P-value
**Number of newborns, n**	251	67	184	
**APGAR score < 7, n/N (%)**	251			
10 minutes		2/67 (3.0%)	5/184 (2.7%)	0.91
**pH at birth, mean ± SD**	219	7.28 ± 0.08	7.26 ± 0.08	0.09
pH < 7,15, n/N (%)	219	5/60 (8.3%)	19/159 (11.9%)	0.45
**Death, n/N (%)**				
Total	251	6/67 (9.0%)	11/184 (6.0%)	0.41
Before 28 days	251	5/67 (7.5%)	8/184 (4.3%)	0.34
After 28 days	251	1/67 (1.6%)	3/184 (1.7%)	0.94
**Neonatal care**				
Intensive care, n/N (%)	251	48/67 (71.6%)	118/184 (64.1%)	0.27
Length of intensive care stay*, days, median (min – max)	149	6 (1 – 63)	6 (1 – 77)	0.12
Lenghts of hospitalisation**, days, median (min – max)	234	15 (3 – 113)	16 (3 – 130)	0.15
**Neonatal complications**				
Respiratory distress, n/N (%)§	249	37/67 (55.2%)	106/182 (58.2%)	0.67
Length of respiratory support**, mean (min – max)	234	10 (0 – 86)	10 (0 – 121)	0.12
Brain abnormalities on MRI, n/N (%) °	251	6/67 (9.0%)	18/184 (9.8%)	0.84
NEC, n/N (%)	251	9/67 (13.4%)	20/184 (10.9%)	0.57
Sepsis, n/N (%)	250	11/67 (16.4%)	40/183 (21.9%)	0.34

**Composite morbi-mortality endpoint, n/N (%) ▫**	251	14/67 (20.9%)	46/184 (25.0%)	0.50

sIUGR: Selective intrauterine growth restriction, IUGR: Intrauterine growth restriction (single pregnancy), SD = standard deviation, NEC: necrotizing enterocolitis. p-value considered statistically significant if < 0.05. *Duration of hospitalization in intensive care unit (ICU) excluding deaths in ICU or newborns not hospitalized in intensive care. **Total duration of hospitalization and duration of respiratory support excluding deaths. § Respiratory distress: Hyaline membrane disease or bronchopulmonary dysplasia. °Brain abnormalities: ventricular dilatation, intraventricular hemorrhage, periventricular leukomalacia, or subependymal hemorrhage. ▫ Composite endpoint defined as the occurrence of one or more of the following events: neonatal death, grade III or IV intraventricular haemorrhage, white matter lesions such as cystic periventricular leukomalacia, bronchopulmonary dysplasia, ulcerative-necrotizing enterocolitis, stage II or III.
Table from L. Gremillet et al [31]

Table S2. Comparison of neonatal status between the “diamniotic monochorionic twin pregnancy with selective intrauterine growth restriction” and “single pregnancy with intrauterine growth restriction”: univariate and multivariate analyses.

	**sIUGR (N = 67)**	**IUGR (N = 184)**	**Univariate analysis**			**Multivariate analysis**		
			**OR**	**95% CI**	P-value	Adjusted OR	95% CI	P-value
**Apgar < 7, n/N (%)**								
10 minutes	2/67 (3.0%)	5/184 (2.7%)	0.91	0.17 – 4.80	0.91	1.06	0.16 – 6.78	0.96
**pH < 7,15, n/N (%)**	5/60 (8.3%)	19/159 (11.9%)	1.49	0.53 – 4.20	0.45	1.51	0.49 – 4.67	0.47
**Intensive care*, n/N (%)**	48/67 (71.6%)	118/184 (64.1%)	0.71	0.38 – 1.30	0.27	0.58	0.23 – 1.45	0.25
**Respiratory distress, n/N (%)**	37/67 (55.2%)	106/182 (58.2%)	1.13	0.64 – 1.99	0.67	1.49	0.66 – 3.36	0.34
**MRI cerebral anomaly, n/N (%)**	6/67 (9.0%)	18/184 (9.8%)	1.10	0.42 – 2.91	0.84	0.93	0.30 – 2.91	0.90
**Visceral complication, n/N (%)**	9/67 (13.4%)	20/184 (10.9%)	0.79	0.34 – 1.82	0.58	0.65	0.23 – 1.85	0.42
**Sepsis, n/N (%)**	11/67 (16.4%)	40/183 (21.9%)	1.42	0.68 – 2.97	0.35	1.24	0.47 – 3.26	0.66
**Neonatal death, n/N (%)**	6/67 (9.0%)	11/184 (6.0%)	0.65	0.23 – 1.82	0.41	0.21	0.04 – 1.04	0.06
**Composite criteria, n/N (%)**	14/67 (20.9%)	46/184 (25.0%)	1.26	0.64 – 2.48	0.50	0.95	0.32 – 2.83	0.92

sIUGR : Selective intrauterine growth restriction, IUGR : intrauterine growth restriction (single pregnancy), OR= odds ratio, 95% CI : confidence interval 95%. P-value considered statistically significant if <0,05. *Intensive care: admission in intensive care unit. Multivariate analysis: adjusted for gestational age at birth, birth weight, fetal gender, birth indication (divided into 5 categories: spontaneous, fetal heart rate abnormalities, fetal doppler abnormalities, growth arrest and other causes) and the last umbilical diastole before birth (positive, null intermittent or permanent). The reference group is sIUGR.

Table from L. Gremillet et al [31]

Table S3a: Comparison of demographic and pregnancy characteristics between the "diamniotic monochorionic twin pregnancy with selective intrauterine growth restriction" and "single pregnancy with intrauterine growth restriction" groups (final population)

	sIUGR	IUGR	P-value
**Number of patients, n**	37	75	
**Mother's age, years, mean ± SD**	29.6 (± 5)	30.4 (±5.4)	0.25
**Gravidity, n, median (min-max)**	2 (1-9)	2 (1-8)	0.67
**Parity, n, median (min-max)**	0 (0-5)	0 (0-4)	0.54
**BMI, kg/m^2, mean ± SD**	23.3 (± 5,1)	25.8 (± 4.9)	0.38
**Socio-professional category, %**			0.38
Not determined	8.1	8	
Unemployed	48.6	30.7	
Management	8.1	9.3	
Intermediate professions	16.2	13.3	
Workers/laborers	16.2	37.3	
Students	2.7	1.3	
**Gestational age at diagnosis, Weeks of amenorrhea, median (min-max)**	27 (12-35)	27 (18-36)	0.06
**Growth discrepancy at diagnosis, %, mean ± SD**	23% ± 8	/	
**Growth percentile at diagnosis, %, mean ± SD**	4.8 (± 5.8)	4.5 (±3.2)	
**Fetal gender, n (%)**			0.45
Male	23 (62.1%)	41 (54.7%)	
Female	14 (37.9%)	34 (45.3%)	
**Antenatal corticosteroid, n/N (%)**			0.06

Not done	2/25 (8%)	4/44 (9.1%)	
Incomplete	0	4/44 (9.1%)	
Complete	23/25 (92%)	36/44 (81.8)	
**Delivery, n (%)**			0.81
Vaginal	11 (30%)	24 (32%)	
**Birth indications, n (%)**			0.03
Spontaneous birth or scheduled caesarean	14 (37.9%)	16 (21.3%)	
Cardiac fetal abnormalities	9 (24.3%)	25 (33.3%)	
Doppler abnormalities	6 (16.2%)	8 (10.7%)	
Growth arrest	4 (10.8%)	15 (20%)	
Pre-eclampsia	2 (5.4%)	11 (14.7%)	
Other	2 (5.4%)	0	
**Gestational age at birth, Weeks of amenorrhea, median (min-max)**	34 (27-38)	33 (26-41)	0.19
**Last umbilical diastole before birth, n (%)**			0.29
Positive	22 (59.5%)	55 (73.3%)	
Null intermittent	7 (18.9%)	11 (14.7%)	
Null permanent	8 (21.6%)	9 (12%)	
**Birth weight, grams, mean ± SD**	1490 ± 490	1511 ± 682	0.29
**Growth discrepancy at birth, %, mean ± SD**	24%	X	X
**Growth percentile at birth, %, mean ± SD**	2.7 ± 2.7	1.6 ± 2.1	0.27
**Percentile of height at birth, %, mean ± SD***	12 ± 10.4	5.7 ± 11	0.24

**Percentile of HC at birth, %, mean ± SD***	16.6 ± 20.8	8.1 ± 11.6	0.33

*data on 97 patients

sIUGR : Selective intrauterine growth restriction, IUGR : intrauterine growth restriction (single pregnancy), HC : head circumference, SD = standard deviation, BMI : body mass index

P-value considered statistically significant if < 0,05

Table S3b: Comparison of neonatal status between the "diamniotic monochorionic twin pregnancy with selective intrauterine growth restriction" and "single pregnancy with intrauterine growth restriction" groups (final population)

	**sIUGR**	**IUGR**	**P value**
**Number of newborns, n**	37	75	
**Apgar score < 7, n (%)**			
10 minutes	1 (2.7%)	2 (2.7%)	0.19
**pH at birth, mean ± SD**	7.3 ± 0.07	7.26 ± 0.09	0.57
pH < 7.15, n (%)	2 (6.3%)	8 (11.9%)	
**Neonatal care**			
ICU, n (%)	29 (78.4%)	41 (54.7%)	
Length of ICU stay*, days, median (min – max)	7 (1 – 51)	10 (1 – 57)	0.13
Length of hospitalization, days, median (min – max)	21 (3 – 91)	20 (3 – 100)	0.58
**Neonatal complications**			
Respiratory distress, n (%)	18 (48.6%)	44 (58.7%)	0.32

Duration of respiratory support, mean (min – max)	10 (0 – 60)	11 (0 – 93)	0.20
Brain abnormalities on MRI, n (%)	5 (13.5%)	8 (10.7%)	0.62
NEC, n (%)	5 (13.5%)	11 (14.7%)	0.78
Sepsis, n (%)	8 (21.6%)	13 (17.3%)	0.58
**Composite morbidity criterion, n (%)**	7 (18.9%)	21 (28.0%)	0.23
**EUGR, n (%)**	25 (67.5%)	52 (69.3%)	0.85

sIUGR: Selective intrauterine growth restriction, IUGR: growth restriction (single pregnancy), SD = standard deviation, NEC: necrotizing enterocolitis, EUGR = extrauterine growth restriction (weight considered insufficient (<5th percentile) at the end of the intensive care/neonatal care unit

P-value considered statistically significant if < 0,05

*Length of stay in intensive care unit (not including neonates never admitted to the intensive care unit)
°Brain abnormalities: ventricular dilation, intraventricular hemorrhage, periventricular leukomalacia, or subependymal hemorrhage
▫ Composite criterion defined as the occurrence of one or more of the following events: neonatal death, grade III or IV intraventricular hemorrhage, cystic periventricular leukomalacia white matter lesions, bronchopulmonary dysplasia, stage II or III ulcerative enterocolitis

Table S4: description of neonatal outcomes of the eutrophic twins

	N	sIUGR	Eutrophic twins	IUGR

**Birth weight, grams, mean ± SD**		1545 ± 567	1964 ± 570	1475 ± 670
**Apgar < 7, n/N (%)**				
10 minutes	318	2/67 (3,0%)	2/67 (3,0%)	5/184 (2,7%)
**pH at birth, mean ± SD**	275	7,28 ± 0,08	7,31 ± 0,07	7,26 ± 0,08
pH < 7,15, n/N (%)	275	5/60 (8,3%)	2/56 (3,6%)	19/159 (11,9%)
**Neonatal deaths, n/N (%)**				
Total	318	6/67 (9,0%)	4/67 (6,0%)	11/184 (6,0%)
Before 28 days	318	5/67 (7,5%)	4/67 (6,0%)	8/184 (4,3%)
After 28 days	318	1/67 (1,6%)	0/67 (0,0%)	3/184 (1,7%)
**Neonatal care**				
Intensive care, n/N (%)	318	48/67 (71,6%)	47/67 (70,1%)	118/184 (64,1%)
Length of time in intensive care unit*, days, median (min – max)	257	6 (1 – 63)	5 (1 – 70)	6 (1 – 77)
Length of time in hospitalization**, days, median (min – max)	297	15 (3 – 113)	14,5 (3 – 113)	16 (3 – 130)
**Neonatal complications**				
Respiratory distress, n/N (%)	316	37/67 (55,2%)	40/67 (59,7%)	106/182 (58,2%)
Duration of respiratory support, mean** (min – max)	297	10 (0 – 86)	9 (0 – 71)	10 (0 – 121)

Brain abnormality on MRI, n/N (%)	318	6/67 (9,0%)	10/67 (14,9%)	18/184 (9,8%)
NEC, n/N (%)	318	9/67 (13,4%)	1/67 (1,5%)	20/184 (10,9%)
Sepsis, n/N (%)	317	11/67 (16,4%)	7/67 (10,4%)	40/183 (21,9%)
**Composite neonatal criterion, n/N (%)** ▫	318	14/67 (20,9%)	11/67 (16,4%)	46/184 (25,0%)

sIUGR: Selective intrauterine growth restriction, IUGR: intrauterine growth restriction (single pregnancy)

*Duration of hospitalization in intensive care not including deaths in intensive care, or newborns not hospitalized in intensive care

**Duration of hospitalization and duration of ventilator support, not including deaths

°Brain abnormalities: ventricular dilatation, intraventricular hemorrhage, periventricular leukomalacia, or subependymal hemorrhage

▫ Composite endpoint defined as the occurrence of one or more of the following events: neonatal death, grade III or IV intraventricular hemorrhage, cystic periventricular leukomalacia white matter lesions, bronchopulmonary dysplasia, stage II or III ulcerative-necrotizing enterocolitis
